# Supplementary material for: Impacts of Air Pollution on Health and Cost of Illness in Jakarta, Indonesia
Source: Int J Environ Res Public Health. 2023 Feb 7;20(4):2916. doi: 10.3390/ijerph20042916 (PMC9963985; doi:10.3390/ijerph20042916)
Supplement: Supplementary file 1 [file ijerph-20-02916-s001.zip › ijerph-2184837-supplementary.pdf]

Impacts of Air Pollution on Health and Cost of Illness in Jakarta, Indonesia

Ginanjar Syuhada[1], Adhadian Akbar[2], Donny Hardiawan[2], Vivian Pun[1], Adi Darmawan[3], Sri Hayyu Alynda Heryati[3], Adiatma Yudistira Manogar Siregar[2], Ririn Radiawati Kusuma[1], Raden Driejana[4], Vijendra Ingole[5], Daniel Kass[5], Sumi Mehta[5,*]

[1] Environmental Health Division, Vital Strategies, Singapore; gsyuhada@vitalstrategies.org
[2] Center for Economics and Development Studies, Department of Economics, Faculty of Economics and Business, Universitas Padjadjaran, Bandung, Indonesia; adiatma.siregar@unpad.ac.id
[3] Environmental Agency of DKI Jakarta Province, Jakarta, Indonesia; darmawanadi@gmail.com
[4] Faculty of Civil and Environment Engineering, Institut Teknologi Bandung, Bandung, Indonesia; driejana@tl.itb.ac.id
[5] Environmental Health Division, Vital Strategies, New York, USA; smehta@vitalstrategies.org
* Correspondence: smehta@vitalstrategies.org

SUPPLEMENTARY

SUPPLEMENTARY 1 – Detailed Definition of Health Outcomes

The following table describes the definition of health outcomes assessed in this study. For the cause-specific mortality, the disease groups are in accordance with the ICD-10 categorization used in the GBD 2019 Study.

Table S1. Summary of health outcomes and their definition included in the study

Outcome	Definition (ICD-10 or otherwise specified)
Children health outcomes	
Infant mortality	All cause
Stunting	Height for age less than two standard deviations of the WHO Child Growth Standards median
Adverse birth outcomes	
Low birth weight, at term	Weight <2,500 g at birth after 37 weeks of gestation
Preterm birth	Birth at the gestation of <37 weeks
Cause-specific mortality	
Ischemic heart disease	I20–I25
Cerebrovascular disease (stroke)	I60–I63, I65–I67, I69.0–I69.3

Chronic obstructive pulmonary disease (COPD)	J40–J44, J47
Type 2 diabetes mellitus	E10–E13
Trachea, bronchus, and lung cancer	C33–C34. D02.1, D02.2, D38.1
Lower respiratory infections	J09–J15.8, J16–J16.9, J20–J21.9, P23-P23.9, and Z25.1

SUPPLEMENTARY 2 – District-specific health burdens

For district-specific health burdens, we found that:

- West Jakarta has the highest $PM_{2.5}$ and O_3 concentrations based on the air quality data from the Environmental Agency (DLH);
- Central Jakarta has the highest rates of adverse health outcomes associated with long term exposure to $PM_{2.5}$ pollution;
- South Jakarta has the highest rate of COPD mortality in the population over 25 associated with long term exposure to O_3 pollution;
- East Jakarta has the highest health burdens (in magnitude) associated with short-term exposure to air pollution, followed by West and South Jakarta.

The following tables describe more detailed information regarding each district-specific health outcome.

Table S2. Infant deaths and stunting cases attributable to long-term exposure to $PM_{2.5}$ in Jakarta districts

		Infant deaths		Stunting	
District	Annual average of $PM_{2.5}$ ($\mu g/m^3$)	Number of cases attributable to $PM_{2.5}$	Rate (per 1000 live births)	Number of cases attributable to $PM_{2.5}$	Rate (per 1000 children under 5)
Central Jakarta	51	80	6	1,072	16
North Jakarta	44	64	3	506	3
West Jakarta	61	104	3	1,561	7
South Jakarta	50	28	1	877	5
East Jakarta	53	53	6	2,233	9

Table S3. Adverse birth outcomes and cause-specific deaths attributable to long-term exposure to $PM_{2.5}$ in Jakarta districts

District	Annual average of $PM_{2.5}$ ($\mu g/m^3$)	Adverse birth outcomes*		Cause-specific deaths^	
		Number of cases attributable to $PM_{2.5}$	Rate (per 1000 live births)	Number of cases attributable to $PM_{2.5}$	Rate (per 100,000 population)
Central Jakarta	51	126	9	1,112	98
North Jakarta	44	225	9	1,410	78
West Jakarta	61	125	4	2,327	92
South Jakarta	50	94	3	2,201	94
East Jakarta	53	154	4	2,641	84

*Combining newborns with low birth weight and babies born preterm

^Total mortality includes deaths from ischemic heart disease, stroke, COPD, type 2 diabetes, lower respiratory infections, and lung cancer.

Table S4. COPD mortality attributable to long-term exposure to Ozone in Jakarta districts

District	Annual mean of daily max 8h-average ($\mu g/m^3$)	COPD Mortality	
		Number of cases attributable to Ozone	Rate (per 100,000 population aged 25+)
Central Jakarta	88	15	2
North Jakarta	125	46	4
West Jakarta	127	70	5
South Jakarta	154	100	7
East Jakarta	127	80	4

Table S5. Numbers of hospital admissions attributable to short-term exposure to $PM_{2.5}$ and Ozone in Jakarta districts

District	Hospital admissions attributable to $PM_{2.5}$		Hospital admissions attributable to Ozone	
	Number of hospitalizations	Rate (per 100,000 population)	Number of hospitalizations	Rate (per 100,000 population aged 65+)
Central Jakarta	635<br>(93% due to CVD; 7% due to RESP)	56	84<br>(90% due to CVD; 10% due to RESP)	143
North Jakarta	465<br>(82% due to CVD; 18% due to RESP)	26	104<br>(78% due to CVD; 22% due to RESP)	140
West Jakarta	820<br>(85% due to CVD; 15% due to RESP)	33	444<br>(92% due to CVD; 8% due to RESP)	405
South Jakarta	322<br>(79% due to CVD; 21% due to RESP)	14	154<br>(95% due to CVD; 5% due to RESP)	136
East Jakarta	1,327<br>(88% due to CVD; 12% due to RESP)	42	414<br>(72% due to CVD; 28% due to RESP)	326

CVD: Cardiovascular diseases;

RESP: Respiratory diseases

SUPPLEMENTARY 3 – Characterizing uncertainties in the health burdens

The following tables describe the lower and upper bounds of confidence intervals from each health burden calculated in this health impact assessment study.

Table S6. Lower- and upper-bound of health outcomes attributable to long-term exposure to air pollution

Health outcomes	DKI Jakarta		Central Jakarta		North Jakarta		West Jakarta		South Jakarta		East Jakarta	
	Lower bound	Upper bound	Lower bound	Upper bound	Lower bound	Upper bound	Lower bound	Upper bound	Lower bound	Upper bound	Lower bound	Upper bound
Associated with PM2.5												
Adverse outcomes in children												
Infant deaths	165	450	40	111	32	90	54	141	14	38	27	73
Stunting	3,987	7,673	694	1,339	319	646	1,041	1,900	565	1,098	1,452	2,777
Low birth weight	302	926	55	169	92	304	55	156	40	123	50	153
Preterm births	88	585	3	23	12	84	9	59	6	38	58	380
Mortality	7,753	11,404	889	1,312	1,109	1,684	1,890	2,716	1,755	2,603	2,119	3,104
Associated with Ozone												
Mortality due to COPD	244	675	8	24	24	70	37	108	54	150	42	124

Table S7. Lower and upper bounds of total hospital admissions attributable to short-term exposure to air pollution

Area	Hospital admissions attributable to PM2.5		Hospital admissions attributable to O3	
	Lower bound	Upper bound	Lower bound	Upper bound
DKI Jakarta	145	14,000	818	2,204
Central Jakarta	72	1,872	44	120
North Jakarta	0*	2,242	50	155
West Jakarta	19	3,429	243	628
South Jakarta	0*	1,788	85	216
East Jakarta	77	4,923	190	630

* The zero value was due to uncertainty in the CI of the RR used in the estimation of health burdens (overlapping 1).

SUPPLEMENTARY 4 – District-specific economic burdens

For district-specific economic burdens, as shown in Table 4, we found that East Jakarta had the highest economic burdens attributable to air pollution in comparison to its GRDP. Mortality burdens contributed to most of the total economic cost attributable to air pollution in each district.

Table S8. The yearly economic cost of health impact attributable to air pollution in DKI Jakarta (in million USD)

District	Health burdens attributable to air pollution					Economic burdens attributable to air pollution						
	Adverse Health Outcomes in Children			Mortality	Hospitalization	Adverse Health Outcomes in Children			Mortality	Hospitalization	**Total economic cost**	**% to GRDP in each district**
	Infant deaths	Stunting	Adverse birth outcomes*			Infant deaths	Stunting	Adverse birth outcomes				
Central Jakarta	80	1,072	126	1,127	719	22.73	0.14	0.19	320.28	0.91	344.25	1%
North Jakarta	64	506	225	1,456	568	18.19	0.06	0.34	413.77	0.68	433.04	2%
West Jakarta	104	1,561	125	2,397	1,264	29.56	0.20	0.04	681.19	1.56	712.54	3%
South Jakarta	28	877	94	2,301	476	7.96	0.11	0.14	653.91	0.57	662.69	2%
East Jakarta	53	2,233	154	2,721	1,472	15.06	0.29	0.22	773.27	2.11	790.94	4%

* Combining newborns with low birth weight and preterm birth
